# Association with Spontaneous Hepatitis C Viral Clearance and Genetic Differentiation of *IL28B/IFNL4* Haplotypes in Populations from Mexico

**DOI:** 10.1371/journal.pone.0146258

**Published:** 2016-01-07

**Authors:** Karina Gonzalez-Aldaco, João R. Rebello Pinho, Sonia Roman, Ketti Gleyzer, Nora A. Fierro, Leticia Oyakawa, Omar Ramos-Lopez, Rubia A. Ferraz Santana, Roberta Sitnik, Arturo Panduro

**Affiliations:** 1 Department of Molecular Biology in Medicine, Civil Hospital of Guadalajara, “Fray Antonio Alcalde”, Guadalajara, Jalisco Mexico and Health Sciences Center, University of Guadalajara, Guadalajara, Jalisco, Mexico; 2 Albert Einstein Medicina Diagnóstica, Hospital Israelita Albert Einstein, São Paulo, SP, Brazil; 3 Laboratorio de Gastroenterologia e Hepatologia Tropical. Instituto de Medicina Tropical, Departamento de Gastroenterologia, Faculdade da Medicina da Universidade de São Paulo, São Paulo, SP, Brazil; 4 Departamento de Patologia Clínica e Anatomia Patológica, Hospital Israelita Albert Einstein, São Paulo, SP, Brazil; University of Sydney, AUSTRALIA

## Abstract

**Aim:**

To analyze the genetic heterogeneity of the Amerindian and admixed population (Mestizos) based on the *IL28B* (rs12979860, rs8099917) and *IFNL4* (rs368234815) haplotypes, and their association with spontaneous clearance (SC) and liver damage in patients with hepatitis C infection from West Mexico.

**Methods:**

A total of 711 subjects from West Mexico (181 Amerindians and 530 Mestizos) were studied for the prevalence of *IL28B* (rs12979860C/T, rs8099917G/T) and *IFNL4* (rs368234815∆G/TT) genotypes. A case-control study was performed in 234 treatment-naïve HCV Mestizos (149 chronic hepatitis C and 85 with SC) for the association of haplotypes with SC and liver damage. A real-time PCR assay was used for genotyping, and transitional elastography staged liver damage.

**Results:**

Significant *Fst-values* indicated differentiation between the studied populations. The frequencies of the protective C, T, TT alleles were significantly lower in the Amerindians than in Mestizos (p<0.05). The r^2^ measure of linkage disequilibrium was significant for all variants and the T/G/ΔG risk haplotype predominated in Amerindians and secondly in Mestizos. The protective C/T/TT haplotype was associated with SC (OR = 0.46, 95% IC 0.22–0.95, p = 0.03) and less liver damage (OR = 0.32, 95% IC 0.10–0.97, p = 0.04) in chronic patients. The Structure software analysis demonstrated no significant differences in ancestry among SC and chronic patients.

**Conclusions:**

West Mexico´s population is genetically heterogeneous at the *IL28B*/*IFNL4* polymorphisms. The T/G/ΔG high-risk haplotype predominated in Amerindians and the beneficial alternative haplotype in Mestizos. The C/T/TT haplotype was associated with SC and less liver damage in chronically infected Mestizo patients.

## Introduction

Hepatitis C virus (HCV) is one of the leading causes of chronic liver disease, affecting more than 150 million people worldwide [1]. Among newly HCV-infected patients, approximately 30% achieve spontaneous clearance (SC) while 70% develop chronic hepatitis C (CHC) infection [2]. CHC may progress to cirrhosis and hepatocellular carcinoma [3]. However, variations in the prevalence of SC suggest the involvement of viral genotypes and host immune factors [4].

Recently, two single nucleotide polymorphisms (SNPs), rs12979860 C/T and rs8099917 G/T within the interleukin-28B (*IL28B*) locus have been associated with SC [5, 6]. These SNPs also confer sustained virological response (SVR) in HCV-infected patients treated with pegylated-interferon-alpha (pegIFN-α) plus ribavirin (RBV) therapy [7]. The *IL28B* gene encodes for interferon lambda-3 (*IFNL3*) that induces a potent antiviral response through JAK-STAT and AMPK pathways [8]. Similarly, Prokunina-Olsson *et al* identified a new gene member of the IFN-λ family, denoted interferon lambda-4 (*IFNL4*), which contains a transiently induced region harboring the dinucleotide variant rs368234815 (ΔG/TT) [9]. IFNL4 is expressed only by ΔG allele carriers and has been considered unfavorable for successful SC and response to pegIFN-α/RBV therapy [9, 10]. After the discovery of *IFNL4*, it became apparent that rs12979860 was located 3 kb upstream of *IFNL3* and was situated in the first intron of *IFNL4*; rs8099917, 9 kb upstream of *IFNL3* and hence outside of *IFNL4* and rs368234815 was in the first exon of *IFNL4* [9].

Genome-wide association studies have shown that the distribution of the *IL28B* (rs12979860, rs8099917) and *IFNL4* (rs368234815) polymorphisms exhibit variations due to ethnicity [6, 9, 11, 12]. For example, several studies in HCV-infected subjects have revealed that the rs12979860CC genotype confers a higher response rate among those with European ancestry than in African-Americans [5, 11]. Likewise, this favorable genotype was found among cohorts of HCV-infected and non-infected subjects in the Spanish population [13] and in HCV-infected US Hispanics [11]. However, one study has reported that the rs12979860CC genotype is more frequent among Egyptians than in Caucasians and even higher among sub-Saharan Africans [14]. Therefore, data regarding these polymorphisms may not be extrapolated to genetically admixed populations, such as the Mexicans or people of other nations of Latin America. Such populations have a genetic architecture characterized by an admixture of three paternal lineages consisting of Amerindian, European and African ancestry, denoted as Mestizos with a heterogeneous distribution within regions or countries [15]. On the other hand, the prevalence of SC in admixed Mexican population has not been estimated and no information exist about the Amerindians population and HCV infection. Moreover, HCV genotype 1 which is known to be associated with non-responsiveness to conventional antiviral therapy is predominant in Mexico and the rest of Latin America [16, 17].

For these reasons, the study of the genetic variations that may affect the course of HCV infection in Latin American populations becomes relevant. This study aimed to analyze the genetic heterogeneity based on the haplotypes of *IL28B* (rs12979860, rs8099917) and *IFNL4* (rs368234815) variants in Mestizos and Amerindian individuals from different regions of West Mexico. We further pursued to analyze the association of the *IL28B/IFNL4* haplotypes with SC and stage of liver damage in treatment-naïve HCV-infected Mestizos patients.

## Materials and Methods

### Study population and design

A comparative cross-sectional study was performed to access the genotypic distribution of *IL28B* (rs12979860, rs8099917) and *IFNL4* (rs368234815) polymorphisms in a total cohort of 711 unrelated individuals. Male and female subjects above 18 years of age were selected from the Amerindian and Mestizos populations at distinct geographic locations of West Mexico. The Amerindian populations were Huicholes (n = 95) from the State of Nayarit and Nahuas (n = 86) from the State of Jalisco. The Mestizos populations were individuals from distinct locations of West Mexico: West-West: city of Tepic, Nayarit (n = 326), Central-West: city of Guadalajara, Jalisco (n = 240), South-West: the town of Villa Purificación (n = 32) (S1 Fig). The Amerindians were members of an ethnic group, spoke their native language and had parents belonging to the ethnic group. The Mestizos subjects were defined as born in Mexico, which spoke Spanish, had Mexican parents and did not belong to any native group [15, 18]. Additionally, a case-control study was carried out to analyze the association between the *IL28B* (rs12979860, rs8099917) *IFNL4* (rs368234815) haplotypes with SC. A total of 234 Mestizos patients newly diagnosed with HCV infection were selected from the Department of Molecular Biology in Medicine of Civil Hospital of Guadalajara “Fray Antonio Alcalde” from 2010 to 2013.

### Ethics

This study protocol was approved by the Ethical Committee of the Hospital Civil of Guadalajara, Guadalajara, Jalisco Mexico and Hospital Israelita Albert Einstein, São Pablo, Brazil. All patients were given an explanation of the study before entering and those who accepted to participate signed an informed written consent. All patients data was handled anonymously and registered using number codes. This study was conducted in compliance with the ethical standards of the 2008 Declaration of Helsinki.

### Clinical evaluation of HCV-infected patients

All patients were treatment-naïve at the time of enrollment. The HCV-infected patients were classified as CHC infection or SC. CHC infection was defined as a positive anti-HCV test and presence of HCV RNA in serum for more than six months. SC was defined as a positive anti-HCV test and absence of HCV RNA in serum at the time of the diagnosis and after a second test six months later. All patients were negative for hepatitis B virus (HBV) and human immunodeficiency virus.

During the initial enrollment, a medical history questionnaire was used to register the amount of alcohol intake. Only patients consuming <20g of alcohol per occasion for women and <40g alcohol per occasion for men (as recommended to prevent liver damage) [19] were included in the study. Alcohol intake was calculated as previously reported [20].

CHC-infected patients were further stratified according to their extent of liver fibrosis measured by transitional elastography (TE) using a Fibroscan^®^ instrument (Echosens, Paris, France). In each patient, liver stiffness (LS) was calculated as the median value of 10 valid TE measurements expressed in kiloPascal (kPa). By this methodology, liver fibrosis is classified as: F1, mild fibrosis (7.1–8.7 kPa), F2, moderate fibrosis (8.8–9.4 kPa), F3, severe fibrosis (9.5–12.4 kPa) and F4, cirrhosis (>12.5 kPa) [21]. In mild cirrhosis (<9.5 kPa, F1 and F2) and cirrhosis (>12.5kPa, F4), LS strongly correlates with the METAVIR score with a 73% sensibility and 91% specificity in the former and 87% sensibility and 91% specificity in the latter [22]. Also, the degree of liver damage in the CHC patients was assessed by the aspartate aminotransferase-to-platelet ratio index (APRI) as previously reported [23].

Anti-HCV antibodies were detected by a third-generation ELISA (AxSYM^®^, Abbott Laboratories, Illinois, USA). HCV viral load was determined by COBAS^®^ AmpliPrep and COBAS^®^ TaqMan^®^ 48 HCV test (Roche Diagnostics, Pleasanton, CA, USA). Liver enzymes aspartate aminotransferase (AST), alanine aminotransferase (ALT) and gamma-glutamyl transpeptidase (GGT) were determined by dry chemistry on a Vitros 250 analyzer (Ortho Clinical Diagnostics, Johnson & Johnson, Rochester, NY, USA). Viral genotyping was performed by a conventional line probe assay (VERSANT HCV Genotype 2.0 Assay LiPA, Siemens Medical Solutions Diagnostics, NY, USA) following the manufacturer’s instructions.

### Ancestry analysis

An estimation of individual ancestry was performed in both SC and CHC patients based on the variations of *IL28B* (rs12979860 and rs8099917) and *IFNL4* (rs368234815). The African, European (HapMap) and Amerindian Huicholes (this study) populations were used as ancestral population references. Genetic ancestry was also assessed by ancestry informative markers (AIMs). For the European ancestry, SNP rs4988235 (C/T -13910) within the lactase (*LCT*) gene was used as previously reported for American admixed populations [24]. The Amerindian ancestry was assessed by using the Apolipoprotein E (*APOE*) gene rs7412 (C/T) because its frequency differs more than 48% between ethnic populations [25, 26].

### Genotyping

Genomic DNA was extracted from peripheral whole blood leukocytes using a salting-out method as previously described [27]. *IL28B* (rs12979860 and rs8099917) and *IFNL4* (rs368234815) loci were genotyped using a 5’ allelic discrimination method. Predesigned TaqMan^®^ SNP Genotyping Assay was used for rs8099917 (C_11710096_10, Applied Biosystems, Foster, CA) City, USA). The rs12979860 and rs368234815 SNPs were tested with custom-designed TaqMan^®^ assays as previously described [9, 11]. The two AIMs were genotyped by using the TaqMan^®^ SNP Genotyping Assays rs4988235, C_2104745_10 for *LCT* and rs7412, C_904973_10 for *APOE* (Applied Biosystems, Foster City, CA, USA). The reactions were carried out in an ABI 7500 Fast Real-Time thermocycler using the standard conditions recommended by the manufacturer. An allelic discrimination plot using the 7500 software (v2.0.6.) automatically attributed the sample’s genotype. Twenty percent of the samples were genotyped twice, and a 100% concordance in genotype allocation was observed. Positive and negative controls were used in each genotyping assay. These genotyping assays were conducted at the Albert Einstein Medicina Diagnóstica, Hospital Israelita Albert Einstein, São Paulo, SP, Brazil.

### Statistical analysis

Genotypic and allelic frequencies were obtained by direct counting method. The categorical variables are expressed as frequency and were compared by chi-square or Fisher’s exact tests. Means of variables were compared with Student’s t-test. Statistical analysis was carried out using the SPSS software (version 20.0) (SPSS, Inc, Chicago, IL). The odds ratio (OR) was calculated with 95% confidence interval using Epi Info^TM^ (7.1.2.0) (Centers for Disease Control and Prevention, Atlanta, GA). Hardy-Weinberg Equilibrium (HWE) and haplotype inference were performed with Arlequin v3 for Windows (Berne, Switzerland). Genetic relatedness between populations and HCV-patients based on *IL28B* and *IFNL4* variations was evaluated by pairwise comparisons (exact tests). Genetic distances (*Fst*-values) were represented in a multidimensional scaling plot (MDS) (SPSS software). Linkage disequilibrium (LD) (r^2^) was calculated by the Genetic Data Analysis (GDA) program (version 1.0.). A p-value <0.05 was considered statistically significant. The estimation of ancestry of the HCV-patients was determined by using maximum likelihood estimation of admixture and Structure software program 2.3.4 [28].

## Results

### Distribution of *IL28B* and *IFNL4* polymorphisms

The distribution of the *IL28B* rs12979860/rs8099917 and *IFNL4* rs368234815 polymorphisms is shown in Table 1. All populations was in HWE (p>0.05) for the three polymorphisms. The Huicholes and Nahuas had lower allelic frequencies for the protective alleles associated with SC than the Mestizos (p<0.05). Conversely, both Amerindian groups had the highest frequency of the respective T, G, ΔG risk alleles for CHC infection. In the Mestizos, the overall frequency of protective alleles for SC was above 58%.

**Table 1:** Allelic and genotypic frequency of *IL28B* and *IFNL4* polymorphisms in West Mexico.

Variant	Huicholes	Nahuas	Guadalajara	Villa Pur	Nayarit	Total WM
**rs12979860**						
**Total N**	95	86	172	32	326	711
C*	84 (44.2)	76 (44.2)	206 (60.0)	48 (75.0)	230 (61.5)	804(56.5)
**T**	106 (55.8)	96 (55.8)	138 (40.0)	16 (25.0)	144 (38.5)	618 (43.5)
**TT**	28 (29.5)	29 (33.7)	22 (12.5)	2 (6.3)	28 (15)	135 (19.0)
**CT**	50 (52.6)	37 (43.0)	93 (54.4)	13 (40.6)	88 (47)	348 (49.0)
**CC**	17 (17.9)	20 (23.3)	57 (33.1)	17 (53.1)	71 (38)	228 (32.0)
**HWE**	0.67	0.27	0.14	1.0	1.0	0.28
**rs8099917**						
**Total N**	95	86	172	32	277	662
**G**	102 (53.7)	96 (55.8)	108 (31.4)	12 (18.7)	116 (42.0)	540(40.8)
T*	88 (46.3)	76 (44.2)	236 (68.6)	52 (81.3)	160 (58.0)	784 (59.2)
**GG**	25 (26.3)	29 (33.7)	14 (8.2)	1 (3.2)	28 (20.3)	112 (17.0)
**GT**	51 (53.7)	37 (43.0)	79 (45.9)	10 (31.3)	61(44.2)	317 (48.0)
**TT**	19 (20.0)	20 (23.3)	79 (45.9)	21 (65.5)	49 (35.5)	233 (35.0)
**HWE**	0.55	0.27	0.47	1.0	0.29	0.27
**rs368234815**						
**Total N**	95	86	167	32	326	706
**ΔG**	106 (55.8)	96 (55.8)	134 (40.0)	16 (25.0)	144 (38.5)	624 (44.2)
TT*	84 (44.2)	76 (44.2)	200 (60.0)	48 (75.0)	230 (61.5)	788 (55.8)
**ΔG/ΔG**	28 (29.5)	30 (34.8)	22 (13.2)	2 (6.3)	29 (15.5)	146 (20.7)
**ΔG/TT**	50 (52.6)	36 (41.9)	91 (54.5)	13 (40.6)	87 (46.5)	332 (47.0)
**TT/TT**	17 (17.9)	20 (23.3)	54 (32.3)	17 (53.1)	71 (38.0)	228 (32.3)
**HWE**	0.67	0.19	0.10	1.0	0.76	0.22

n/N, Number. Allelic and genotypic frequencies were 2n (%) or n (%), respectively. Variations in the number of individuals were due to the lack of sample availability.

* The pooled prevalence of Amerindians alleles *vs*. pooled prevalence of Mestizos alleles was significant (p<0.05). Villa Pur, Villa Purificación; WM, West Mexico

### Distribution of *IL28B* and *IFNL4* haplotypes and genetic relationship among populations

The degree of LD of the *IL28B* and *IFNL4* polymorphisms varied among the studied populations (S1 Table). *IL28B* rs12979860 polymorphism was in high LD (r^2^ = 0.72–1) with *IFNL4* rs368234815 in all populations, while the *IL28B* rs8099917 polymorphism was in moderate LD with the rs368234815 (*IFNL4*) variant, except in the Nahuas population (r^2^ = 0.92).

The distribution of five haplotypes found in this study is shown in Table 2. The most frequent combination among the Mestizos was the C/T/TT haplotype (*IL28B* rs12979860/*IL28B* rs8099917/*IFNL4* rs368234815) containing the protective alleles associated with SC. Notably, the population of Villa Purificación had the highest frequency of this haplotype (73.5%). Conversely, the T/G/∆G haplotype containing the risk alleles associated with CHC infection was the most frequent (>55%) among Huicholes and Nahuas and was the second most prevalent in the Mestizo populations (19–30%). The remaining three haplotypes had frequencies of <16%.

**Table 2:** Haplotype frequency of *IL28B* and *IFNL4* polymorphisms in West Mexico.

Population	N	Haplotype n (%)				
		C/T/TT	T/G/ΔG	T/T/ΔG	C /T/ΔG	C/G/TT
**Huicholes**	**95**	43 (45.0)	52 (55.0)	-	-	-
**Nahuas**	**86**	38 (44.0)	48 (56.0)	-	-	-
**Guadalajara**	**167**	97 (58.0)	50 (30.0)	13 (8.0)	3 (1.5)	-
**Nayarit**	**277**	116 (42.0)	72 (26.0)	43 (15.5)	-	39 (14.0)
**Villa Purificación**	**32**	24 (73.5)	6 (19.0)	2 (7.5)	-	-
**Total WM**	**657**	**315 (48.0)**	**230 (35.0)**	**57 (8.6)**	**-**	**36 (5.4)**

n/N, Number; WM, West Mexico. Allele order: rs12979860/rs8099917/rs368234815. Only haplotypes with frequency >1% were reported.

**Fig 1:**
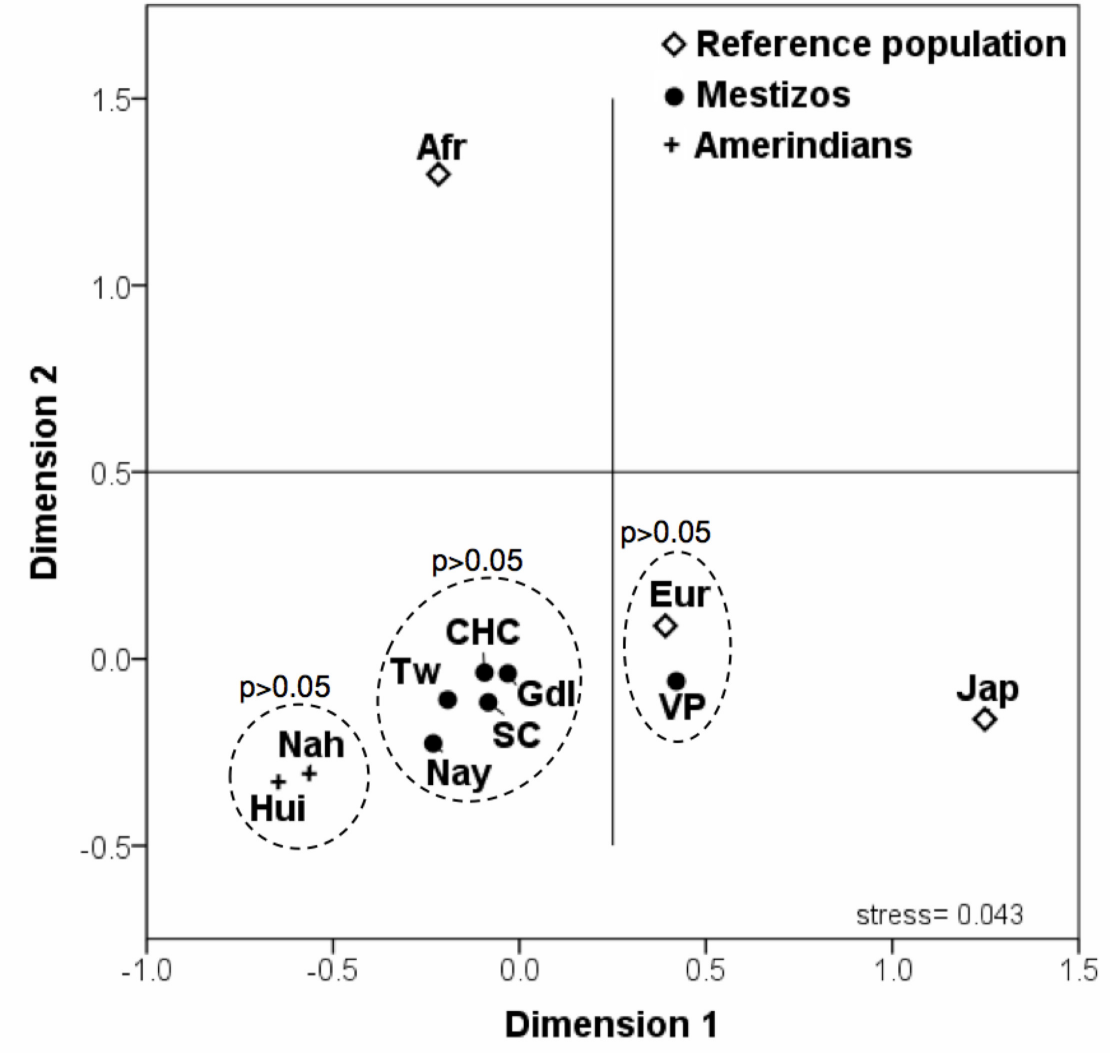
Multidimensional scaling (MDS) plot based on genetic differentiation (*Fst*) values between Mexicans and reference populations. Nah, Nahuas; Hui, Huicholes; Nay, Nayarit; TW, Total West Mexico; Gdl, Guadalajara; VP, Villa Purificación; Cau, Caucasians; Jap, Japan and Afr, Africa; SC, spontaneous clearance; CHC, chronic hepatitis C.

Genetic differentiation was measured by *Fst*-values (S2 Table). For comparative purposes, the frequencies of African, Caucasian and Japanese populations reported in the HapMap Project were included as references [26]. Three clusters were generated from the studied populations (p>0.05) (Fig 1). The individuals from Villa Purificación, Jalisco formed a cluster on the lower right side of the plot together with the Caucasian reference subjects, whereas the Amerindians, Huicholes and Nahuas grouped in the lower left corner. As expected, the Mestizos from Guadalajara and the total West Mexico data formed an intermediate cluster.

### Association of *IL28B* and *IFNL4* polymorphisms with clinical outcome of HCV infection and liver damage

Among a total of 234 patients, 63.6% (n = 149) had CHC infection and 36.4% (n = 85) presented SC. The clinical and demographic characteristics are shown in Table 3.

**Table 3:** Demographic and clinical characteristics of HCV-infected patients.

Variable	Chronic Infection	Spontaneous Clearance	*P-value*
**Number of subjects**	149	85	
**Age (years)**	49.4 ± 12.5	46.8 ± 13.9	*NS*
**Male n (%)**	57 (38.2)	37 (43.5)	*NS*
**Female n (%)**	92 (61.7)	48 (56.5)	*NS*
**BMI (kg/m**^**2**^**)**	26.5 ± 5.6	27.9 ± 4.1	*NS*
**AST (IU/L) (Median, interquartil range)**	60.5 (40–94.2)	27.5 (23–35)	*1*.*0x10*^*-13*^
**ALT (IU/L) (Median, interquartil range)**	62.0 (34.7–102.5)	24.0 (19–35)	*1*.*9x10*^*-14*^
**Hemogloblin (g/dL)**	13.8 ± 2.0	14.6 ± 1.7	*0*.*01*
**Platelets (x10**^**3**^**/μl)**	160.5 ± 87.6	226.5 ± 78.2	*1*.*2x10*^*-5*^
**HCV load (x10**^**6**^ **IU/mL)**	6.21 ± 9.83	Undetectable	
**Viral genotypes**^**a**^ **n (%)**			
**1**	2 (1.8)	NA	
**1a**	54 (47.8)	NA	
**1b**	17 (15.0)	NA	
**2**	16 (14.2)	NA	
**2a/2c**	3 (2.7)	NA	
**2b**	8 (7.0)	NA	
**3**	1 (0.9)	NA	
**3a**	12 (10.6)	NA	
**Duration of infection (years)**	27.16 ± 13.7	22.0 ± 12.1	*NS*
**Ancestry**			
**Amerindian (%)**	27.1	28.6	*0*.*354*
**European (%)**	66.7	68.0	*0*.*840*
**African (%)**	6.2	3.4	*0*.*802*

Data are expressed as mean ± SD, unless specified. N, number

^a^HCV genotype was available for 113 individuals in the chronic infection group. BMI, body mass index; AST, aspartate aminotransferase; ALT, alanine aminotransferase; NA, not applicable; NS, not significant.

The mean age was 49.4 ± 12.5 years for the CHC patients and 46.8 ± 13.9 in the SC group. No differences in age, gender and body mass index were found between groups. Higher levels of AST and ALT enzymes were found in the patients with CHC infection than in those with SC (p<0.05). The CHC patients were mainly carriers of HCV genotype 1a (47.8%) and 1b (15%) followed by genotypes 2 and 3. The duration of infection among HCV-patients was estimated by the self-reported date of exposure to known risk factors for HCV infection, which were mainly blood transfusion, surgery, tattoos and injecting drug use [16]. When the HCV-patients were compared by ancestry contribution, according to *IL28B* and *IFNL4* genotypes, the Structure software analysis identified three well-differentiated populations (κ = 3). The European, African and Amerindian ancestry was similar in both the SC and CHC patients (p<0.05). Also, the frequency of two AIMs (rs4988235 and rs7412) was not significantly different between the SC and HCV patients (S3 Table).

Moreover, the CHC patients were stratified by stage of liver damage (Table 4). Significant differences in the indicators of liver damage were observed between stages F1 and F4. However, there were no differences in the duration of infection between CHC patients with mild and severe fibrosis (F1-F2, 26.7 ± 13.7 *vs*. F3-F4, 27.6 ± 14.0 years, p = 0.7).

**Table 4:** Clinical characteristics by degree of fibrosis in CHC patients.

Variable	F1	F2	F3	F4
**Number of subjects**	39	8	9	28
**Age (years)**	45.6 ± 14.2^a^	46.7 ± 11.1	51.3 ± 13.5	54.8 ± 11.1
**Female, n (%)**	20 (51.2)	4 (50)	6 (66.7)	22 (78.5)
**Liver stiffness (kPa)**	5.6 ± 1.4^b^^c^^d^	9.1 ± 0.3^e^^f^	11.9 ± 0.4 ^g^	30.6 ± 18.0
**APRI score**	0.46 ± 0.5^h^	0.8 ± 0.9	1.4 ± 1.6	1.5 ± 1.1
**AST (IU/L)**	37.9 ± 17.6^i^	65.1 ± 50.0	75.6 ± 64.4	80.8 ± 50.8
**ALT (IU/L)**	48.3 ± 32.3^j^	68.5 ± 45.9	66.25 ± 62.4	86.3 ± 59.8
**GGT (IU/L)**	41.9 ± 54.0^k^	23.6 ± 9.9^l^	87.2 ± 110.3	100.9 ± 106
**Alkaline phosphatase (IU/L)**	84.0 ± 26.0^m^	83.8 ± 29.3^n^	103.0 ± 28.4	137.9 ± 77.4
**Platelets, x10**^**3**^**/μl**	225.5 ± 76.8^o^	212.6 ± 60.8	141.9 ± 55.0	158.9 ± 84.4
**Albumin, g/dl**	4.0 ± 0.4^p^	4.0 ± 0.6	4.1 ± 0.3	3.6 ± 0.5

Data were expressed as mean ± SD. kPa, kilopascal; APRI, aspartate aminotransferase-to-platelet ratio index; AST, aspartate aminotransferase; ALT, alanine aminotransferase; GGT, gamma-glutamyl transpeptidase.

^a^F1 *vs*. F4 *p = 0*.*01*

^b^F1 *vs*. F2 *p = 3*.*6x10*^*-7*^

^c^F1 *vs*. F3 *p = 1*.*8x10*^*-8*^

^d^F1 *vs*. F4 *p = 4*.*3x10*^*-7*^

^e^F2 *vs*. F3 *p = 0*.*001*

^f^F2 *vs*. F4 *p = 1*.*4x10*^*-6*^

^g^F3 *vs*. F4 *p = 2*.*9x10*^*-7*^

^h^F1 *vs*. F4 *p = 0*.*001*

^i^F1 *vs*. F4 *p = 4*.*1x10*^*-3*^

^j^F1 *vs*. F4 *p = 0*.*014*

^k^F1 *vs*. F4 *p = 0*.*046*

^l^F2 *vs*. F4 *p = 0*.*003*

^m^F1 *vs*. F4 *p = 0*.*003*

^n^F2 *vs*. F4 *p = 0*.*017*

^o^F1 *vs*. F4 *p = 0*.*005*

^p^F1 *vs*. F4 *p = 0*.*001*

Table 5 depicts the OR (95% CI) of the association between the individuals *IL28B* and *IFNL4* polymorphisms and SC. Only rs12979860 and rs368234815 were associated with SC, whereas rs8099917 was not associated. Notably, the SC and CHC patients showed no differences in the *Fst-values* when compared to the other Mestizo populations (Gdl and Nay) as shown in Fig 1.

**Table 5:** Association of *IL28B* and *IFNL4* polymorphisms on the clinical outcome of HCV infection.

Variant	Chronic infection (n,%)	Spontaneous clearance (n,%)	*p-value*	Comparison	OR (95% CI)	*p-value*
***IL28B* rs12979860**						
**CC**	35 (23.5)	39 (45.8)	*1*.*4x10*^*-3*^	CC *vs*. TT	0.44 (0.20–0.96)	*0*.*03*
**CT**	84 (56.4)	31 (36.0)	*3*.*4x10*^*3*^	CC *vs*. CT	0.33 (0.17–0.61)	*3*.*4x10*^*-4*^
**TT**	30 (20.1)	15 (18.6)	*0*.*64*	CC *vs*. CT+TT	0.36 (0.20–0.64)	*3*.*9x10*^*-4*^
**Total**	**149 (100)**	**85 (100)**				
***IL28B* rs8099917**						
**GG**	12 (11.0)	8 (13.0)	*0*.*78*	TT *vs*. GG	1.08 (0.39–2.90)	*0*.*87*
**GT**	52 (48.0)	29 (45.0)	*0*.*71*	TT *vs*. GT	0.90 (0.46–1.75)	*0*.*77*
**TT**	44 (41.0)	27 (42.0)	*0*.*85*	TT *vs*. GT+ GG	0.62 (0.34–1.15)	*0*.*13*
Total*	**108 (100)**	**64 (100)**				
***IFNL4* rs368234815**						
**ΔG/ΔG**	23 (20.0)	8 (12.0)	*0*.*17*	TT/TT *vs*. ΔG/ΔG	0.38 (0.16–0.89)	*0*.*02*
**ΔG/TT**	62 (54.0)	29 (44.0)	*0*.*19*	TT/TT *vs*. ΔG/TT	0.49 (0.24–0.95)	*0*.*03*
**TT/TT**	30 (26.0)	29 (44.0)	*0*.*01*	TT/TT *vs*. ΔG/ΔG + ΔG/TT	0.45 (0.23–0.85)	*0*.*01*
Total*	**115 (100)**	**66 (100)**				

n, number.

* Due to lack of sample availability, only 172 and 181 patients were tested for rs8099917 and rs368234815, respectively.

A dominant genetic model was used to assess the association of the *IL28B/IFNL4* haplotypes with SC and liver damage. The C/T/TT haplotype was associated with SC (C/T/TT *vs*. T/G-T/∆G; OR = 0.46, 95% IC 0.22–0.95 p = 0.03) (Table 6). Additionally, the association between the haplotype frequency among the CHC patients with liver damage (F1 *vs*. F4) showed that the C/T/TT haplotype carriers had less liver damage compared to the T/G/∆G haplotype carriers (OR = 0.32, 95% IC 0.10–0.97, p = 0.04) (Table 7).

**Table 6:** Association of the *IL28B* and *IFNL4* haplotypes with clinical outcome of HCV infection.

Haplotype	Chronic infection (N = 115)	Spontaneous Clearance (N = 66)	*p- value*	Comparison	OR (95% CI)	*p-value*
**C/T/TT (n, %)**	51 (44.5)	36 (53.8)	*0*.*03*			
**T/G/ΔG (n, %)**	31 (26.9)	15 (22.7)	*0*.*75*	C/T/TT *vs*. T/G/ΔG + T/T/ΔG	0.46 (0.22–0.95)	*0*.*03*
**T/T/ΔG (n, %)**	15 (12.6)	-	*3*.*1x10*^*-3*^			

N/n, Number. Allele order: rs12979860/rs8099917/rs368234815. Only complete haplotypes in each group were considered for analysis. Only haplotypes with frequency >1% were reported.

**Table 7:** Association of the *IL28B* and *IFNL4* haplotypes with stage of liver damage in CHC patients.

Haplotype	Low damage (F1) (N = 39)	Cirrhosis (F4) (N = 28)	*p-value*	Comparison	OR (95% CI)	*p-value*
**C/T/TT (n, %)**	23 (59.0)	13 (48.2)	*0*.*04*			
**T/G/ΔG (n, %)**	6 (16.6)	12 (43.0)	*0*.*03*	C/T/TT *vs*. T/G/ΔG + T/T/ΔG	0.32 (0.10–0.97)	*0*.*04*
**T/T/ΔG (n, %)**	2 (6.0)	2 (7.0)	*0*.*52*			

N, Number; Allele order: rs12979860/rs8099917/rs368234815. Only complete haplotypes in each group were considered for analysis. Only haplotypes with frequency >1% were reported.

## Discussion

In this study, the *IL28B* rs12979860/rs8099917 and *IFNL4* rs368234815 alleles, genotypes, and their respective haplotypes showed different frequency distributions between Amerindian and Mestizo populations. The lowest frequency of the individual C, T, TT alleles was found in the Amerindians and the highest in Mestizos (p<0.05). This data is in agreement with the historical, genetic and cultural backgrounds of each study group [29, 30]. To our knowledge, this is the first study regarding the genetic features of the rs8099917 and rs368234815 polymorphisms in Amerindian populations from Mexico. In this study, the Huicholes and Nahuas exhibited a greater frequency of the ancestral rs368234815 ∆G allele. Conversely, the Mestizos populations showed the highest prevalence of the respective C, G and TT alleles known to be associated with SC. These frequencies are consistent with those reported by others in Central Mexico, which are similar to Mexican-Americans and Caucasians [31–33]. Thus, given this pattern of genetic structure, it is plausible that this may also occur in other native and admixed groups throughout Latin America [34], although further studies are needed to evaluate this assumption.

Given the close location of the *IL28B* (rs12979860, rs8099917) and *IFNL4* (rs368234815) polymorphisms, several studies have been performed to calculate the levels of LD between the two genes [9,35, 36]. In this study, a high LD (r^2^ = 0.72–1) between rs12979860 and rs368234815 was found in all studied populations. However, lower LD values were found among the Mestizos for rs8099917 and rs368234815 compared to the Nahuas. Thus, when inferring the haplotypes, we found an overall heterogenic distribution. The T/G/∆G haplotype (rs12979860 /rs8099917/rs368234815) was the most frequent among the Amerindians, whereas the C/T/TT haplotype was higher in the Mestizos. Among these, the population from Villa Purificación showed the highest frequency of this haplotype, which is consistent with data previously reported in regards to their strong European ancestry [20]. Furthermore, the frequencies of the protective haplotype C/T/TT observed in the Mestizos and HCV-infected patients from Guadalajara were both similar to those reported in Caucasians [9]. Thus, it is plausible that the European genetic component that exists in the Mestizos populations from West Mexico favors the predominance of C/T/TT haplotype [15]. On the other hand, the high frequency of the T/G/∆G haplotype in the Amerindians was consistent with the allelic and genotypic frequencies found in this study. These findings may also explain why it is the second most frequent in the Mestizos populations, due to the admixture of European and Amerindian ancestry [15].

Moreover, it has been reported that SC is influenced by host factors [37]. Among these, the immune response plays a critical role in SC and the development of liver damage in HCV patients [38]. In this study, the rs12979860CC and rs368234815TT/TT genotype were associated with SC in Mestizos patients of West Mexico, which is in agreement with previous research [9, 39–41]. However, to the best of our knowledge, the association of rs368234815TT/TT genotype with SC has not been previously reported among HCV patients in Mexico and Latin America. Furthermore, the association of this polymorphism with SC is maintained among the admixed population with Caucasian ancestry and in less proportion in the Amerindians. Also, the genetic ancestry was equally distributed among the SC and CHC patients. Thus, the association with SC was independent of the admixture among the study groups. In regards to the socio-economic factors that may influence the clinical outcome, the CHC patients attending the hospital have an urban residence, belong to a middle-income status and are exposed to the same environmental factors. On the other hand, no association was found with the rs8099917TT genotype and SC. This result is consistent with a report in Chinese patients [35] and with one previous study in Central Mexico that found no association with the response to pegIFN-α/RBV therapy [32]. In concordance with these observations, in this study the C/T/TT haplotype was associated with SC and less liver damage than those with the risk haplotype.

The mechanism by which these alleles are associated with SC or chronic infection remains unclear. For example, micro RNA-122 expression in rs12979860 CT/TT carriers had been related to liver fibrosis [42]. However, it is plausible that it may be linked to different immune response profiles. Studies *in vitro* have shown that IFNL4 induces expression of CCL5 (RANTES), a molecule considered a biomarker of early-stage liver damage [9, 10, 43, 44]. This could be the reason patients carrying the ∆G allele present greater liver damage. Furthermore, this hypothesis is consistent with recent findings that demonstrated that chronic HCV-infected patients express higher levels of interleukin-8 and RANTES in comparison with those who had SC [45]. Nonetheless, the impact of *IL28B* and *IFNL4* polymorphisms on HCV-related disease progression is controversial [46–49]. The discrepancy in disease outcome reported worldwide may be due to differential immunogenetic responses based on ethnic and environmental factors, such as diet, physical activity, obesity and other associated co-morbidities [37, 50].

Among the Old World human populations, positive selection may be responsible for the increased frequency of the protective rs368234815TT allele [51]. However, the presence of the TT allele among the Mestizo group may be due to the recent introduction of the European component into the New World Amerindian population (some 500 years ago) [50] combined with the exposure to diverse viral agents. In contrast, the high frequency of the ancestral ∆G allele and haplotypes in Amerindians mirrors that of non-admixed human populations. Therefore, in regards to the Mexican population, it is plausible that carriers of the ∆G allele regardless of the Mestizo or Amerindian ancestry could have a more complicated clinical outcome if they were infected with HCV. Furthermore, HCV genotypes 1a and 1b are the most predominant in the country and pegIFN-α/RBV is the conventional antiviral treatment [52, 53]. Thus the likelihood of SVR decreases and significant adverse effects may occur [54]. However, further studies are needed to test these hypotheses.

Interestingly, the Amerindian groups in Mexico appear to be protected against the progression of HBV infection [50, 55]. Nonetheless, distinct mechanisms may be involved in the progression of HBV and HCV in this population. Although *IL28B* polymorphism does not predict long-term response to interferon therapy in HBeAg-positive chronic hepatitis B patients [56], previous reports have shown that a cytokine expression profile evoked by HBV infection is different between Amerindians and Mestizos population [55]. Thus, it is liable to expect immune-based differences against HCV in these groups. On the other hand, it is of notice that the Amerindian populations throughout Latin America are endemic for HBV infection [57] and, it is accepted that HBV/HCV co-infections cause more severe liver damage [58]. Consequently, there is an urgent need for improving our understanding of the mechanisms involved in the progression of the infections associated with these etiological agents. Furthermore, the impact of the *IL28B*/*IFNL4* haplotypes requires to be evaluated in the context of the current therapies available for handling CHC patients.

On the other hand, it has been suggested that metabolic factors could be playing a role in the clinical outcome of HCV infection [37]. *IL28B* polymorphisms have been related to the content of serum lipids [59, 60]. In this context, the Mexican population is currently in a high state of metabolic disorders and obesity, which are accompanied by insulin resistance a common characteristic of HCV infection [61, 62]. However, further studies are needed to evaluate the impact of this condition on the outcome of HCV infection among our population.

Finally, our data creates the basis to develop strategies regarding personalized medicine for the Mexican population to minimize side effects and to maximize treatment success [63]. Moreover, they highlight the need to study these polymorphisms in other admixed populations in Latin America and abroad. The C/T/TT haplotype could be a powerful tool to predict SC and response to therapy and potentially to enable physicians to select better candidates for antiviral treatment.

In conclusion, a heterogeneous genotypic distribution of the *IL28B*/*IFNL4* polymorphisms among Mestizos and Amerindian populations from West Mexico was evident. The clinical outcome of CHC infection in Mexican Mestizos may be influenced by the C/T/TT and T/G/∆G haplotypes, whereas the potential T/G/∆G high-risk haplotype predominated in Amerindians.

## Supporting Information

S1 Fig Geographical distribution of West Mexican Populations. VP, Villa Purificación; HCV, hepatitis C virus (TIF).
(TIF)

S1 Table S1 Table (PDF). (PDF)

S2 Table S2 Table (PDF). (PDF)

S3 Table S3 Table (PDF). (PDF)
