# Supplementary figures and images for: Association with Spontaneous Hepatitis C Viral Clearance and Genetic Differentiation of IL28B/IFNL4 Haplotypes in Populations from Mexico
Source: PLoS One. 2016 Jan 7;11(1):e0146258. doi: 10.1371/journal.pone.0146258 (PMC4704808; doi:10.1371/journal.pone.0146258)

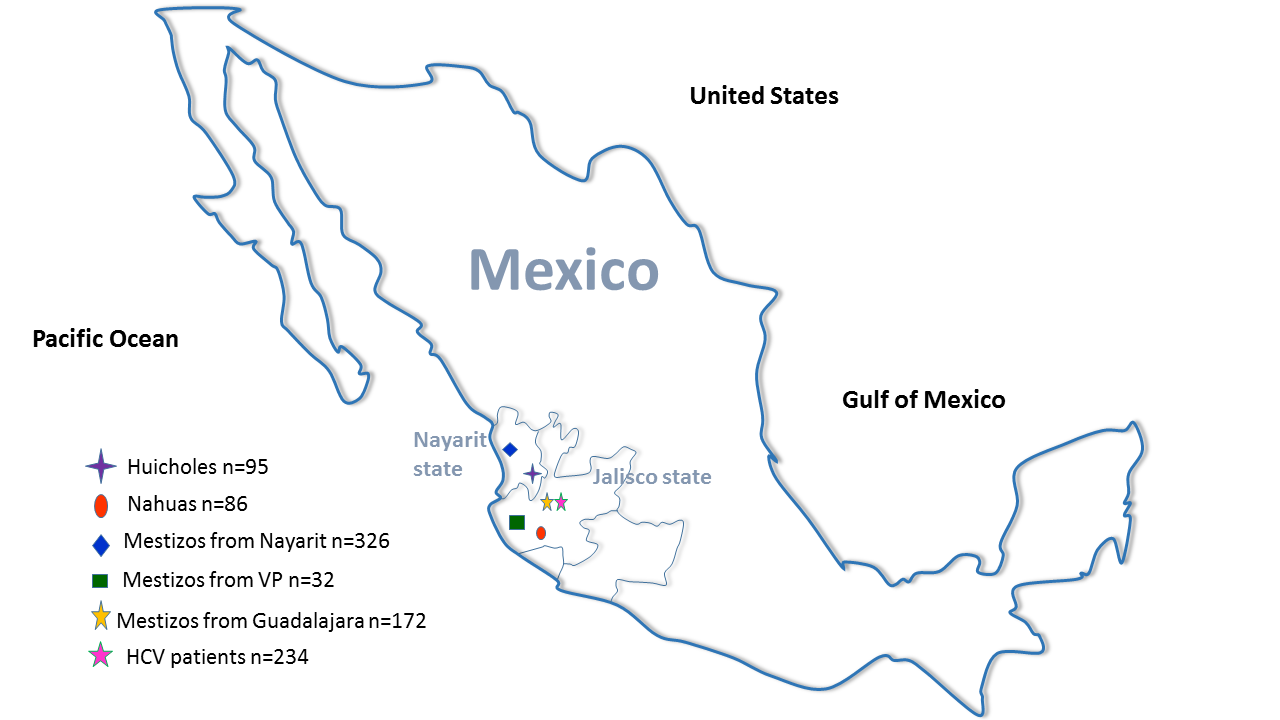

Supplement: S1 Fig — VP, Villa Purificación; HCV, hepatitis C virus (TIF). (TIF) [file pone.0146258.s001.tif]
