# Supplementary material for: Association with Spontaneous Hepatitis C Viral Clearance and Genetic Differentiation of IL28B/IFNL4 Haplotypes in Populations from Mexico
Source: PLoS One. 2016 Jan 7;11(1):e0146258. doi: 10.1371/journal.pone.0146258 (PMC4704808; doi:10.1371/journal.pone.0146258)
Supplement: S1 Table — (PDF) [file pone.0146258.s002.pdf]

Supplementary Table 1. Analysis of Linkage Disequilibrium of *IL28B* and *IFNL4* polymorphisms in West Mexico populations

Population	Marker	Distance (bp)	r^2	Allele	Allele frequency (%)	Number of Chromosomes
**HCV-infected patients**	rs368234815	0	1	TT	57.8	362
	rs12979860	367	0.72	C	56.1	362
	rs8099917	3946	0.55	T	64.8	362
**Guadalajara**	rs368234815	0	1	TT	59.6	334
	rs12979860	367	0.92	C	60.2	334
	rs8099917	3946	0.78	T	68.9	334
**Nayarit**	rs368234815	0	1	TT	61.2	554
	rs12979860	367	0.94	C	61.5	554
	rs8099917	3946	0.45	T	57.6	554
**Huicholes**	rs368234815	0	1	TT	44.2	190
	rs12979860	367	1	C	44.2	190
	rs8099917	3946	0.7	G	46.8	190
**Nahuas**	rs368234815	0	1	TT	44.2	172
	rs12979860	367	0.92	C	44.8	172
	rs8099917	3946	0.92	T	44.8	172
**Villa Purificación**	rs368234815	0	1	TT	73.4	64
	rs12979860	367	1	C	73.4	64
	rs8099917	3946	0.8	T	81.2	64
**Total West Mexico**	rs368234815	0	1	TT	55.8	1314
	rs12979860	367	0.96	C	56.5	1314
	rs8099917	3946	0.68	G	59.1	1314

Only complete haplotypes by population were considered for the analysis. The distance (bp) is given with respect to rs368234815 according to Prokunina-Olsson *et al* [9].
