# Supplementary material for: Association with Spontaneous Hepatitis C Viral Clearance and Genetic Differentiation of IL28B/IFNL4 Haplotypes in Populations from Mexico
Source: PLoS One. 2016 Jan 7;11(1):e0146258. doi: 10.1371/journal.pone.0146258 (PMC4704808; doi:10.1371/journal.pone.0146258)
Supplement: S2 Table — (PDF) [file pone.0146258.s003.pdf]

Supplementary Table 2. Pairwise *Fst* distances (below diagonal), and *Fst P-values* (above diagonal) among Mexican Mestizos, Natives, HCV-patients and references populations

	**Tw**	**Afr**	**Jap**	**Eur**	**VP**	**Gdl**	**Nay**	**Hui**	**Nah**	**SC**	**CHC**
**Tw**	-	***0.00000***	***0.00000***	***0.00000***	***0.00000***	0.07207	0.32432	**0.01802**	**0.00901**	0.27027	0.09910
**Afr**	0.22195	-	***0.00000***	***0.00000***	***0.00000***	***0.00000***	***0.00000***	***0.00000***	***0.00000***	***0.00000***	***0.00000***
**Jap**	0.23119	0.46245	-	***0.00000***	***0.00000***	***0.00000***	***0.00000***	***0.00000***	***0.00000***	***0.00000***	***0.00000***
**Eur**	0.09585	0.20641	0.11288	-	0.86486	***0.00000***	***0.00000***	***0.00000***	***0.00000***	***0.00000***	***0.00000***
**VP**	0.09804	0.29784	0.06847	0.000000	-	***0.03604***	***0.00000***	***0.00000***	***0.00000***	***0.00901***	***0.00000***
**Gdl**	0.02174	0.20478	0.20792	0.04001	0.05137	-	***0.02703***	***0.00000***	***0.00000***	0.55856	0.73874
**Nay**	0.01189	0.32598	0.28589	0.12965	0.10271	0.02938	-	***0.00901***	***0.04306***	0.33333	***0.02703***
**Hui**	0.04366	0.34616	0.45448	0.26080	0.23265	0.09317	0.05068	-	0.66667	***0.02703***	***0.00000***
**Nah**	0.03210	0.34290	0.42574	0.23660	0.20741	0.07766	0.03504	0.00389	-	***0.01802***	***0.00000***
**SC**	0.01353	0.25701	0.22553	0.06364	0.05939	0.00777	0.01306	0.07749	0.06082	-	0.47748
**CHC**	0.01693	0.19391	0.22339	0.05732	0.06878	0.00754	0.02618	0.07472	0.06165	0.00933	-

Significant *Fst P-values* are in bold (<0.05), Tw: Total West, Afr: African, Jap: Japan, Eur: European, VP: Villa Purificación, Gdl: Guadalajara, Nay: Nayarit, Hui: Huicholes, Nah: Nahuas, SC: spontaneous clearance, CHC: Chronic hepatitis C
