# Supplementary material for: Association with Spontaneous Hepatitis C Viral Clearance and Genetic Differentiation of IL28B/IFNL4 Haplotypes in Populations from Mexico
Source: PLoS One. 2016 Jan 7;11(1):e0146258. doi: 10.1371/journal.pone.0146258 (PMC4704808; doi:10.1371/journal.pone.0146258)
Supplement: S3 Table — (PDF) [file pone.0146258.s004.pdf]

Supplementary Table 3. Allele frequency of the AIMs in ancestral populations and HCV-patients

Markers	Reference Populations			HCV-patients		
	**European**	**Amerindian**	**African**	**SC**	**CHC**	***p-value***
**LCT*	0.73	0.01	.00	0.043	0.043	1.00
**APOE*	0.50	1.00	.90	0.890	0.913	0.99

*T allele; AIMs, Ancestry Informative Markers; SC, spontaneous clearance; CHC, chronic hepatitis C; *LCT,* lactase; *APOE,* apolipoprotein E.
